# Elucidating the role of dsRNA sensing and Toll6 in antiviral responses of *Culex quinquefasciatus* cells

**DOI:** 10.3389/fcimb.2023.1251204

**Published:** 2023-08-30

**Authors:** Brian C. Prince, Kalvin Chan, Claudia Rückert

**Affiliations:** Department of Biochemistry and Molecular Biology, College of Agriculture, Biotechnology & Natural Resources, University of Nevada, Reno, NV, United States

**Keywords:** mosquito, *Culex*, immune sensing, dsRNA sensing, Toll, arbovirus, La Crosse virus

## Abstract

The first step of any immune response is the recognition of foreign molecular structures inside the host organism. An important molecule that is generally foreign to eukaryotic cells is long double-stranded RNA (dsRNA), which can be generated during virus replication. The mechanisms of sensing viral dsRNA are well-studied in mammalian systems but are only poorly understood in insects, including disease vectors such as *Culex quinquefasciatus* mosquitoes. These mosquitoes are vectors for important arboviruses, such as West Nile virus, and *Culex* species mosquitoes are distributed across the globe in many temperate and tropical regions. The major antiviral response triggered by dsRNA in mosquitoes is RNA interference – a sequence-specific response which targets complementary viral RNA for degradation. However, here, we aimed to identify whether sequence-independent dsRNA sensing, mimicked by poly(I:C), can elicit an antiviral response. We observed a significant reduction in replication of La Crosse virus (LACV) in *Cx. quinquefasciatus* mosquito cells following poly(I:C) priming. We identified a number of antimicrobial peptides and Toll receptors that were upregulated at the transcript level by poly(I:C) stimulation. Notably, Toll6 was upregulated and we determined that a knockdown of Toll6 expression resulted also in increased LACV replication. Future efforts require genetic tools to validate whether the observed Toll6 antiviral activity is indeed linked to dsRNA sensing. However, large-scale functional genomic and proteomic approaches are also required to determine which downstream responses are part of the poly(I:C) elicited antiviral response.

## 1 Introduction

Mosquito-borne diseases have been historically localized to tropical and subtropical regions of the world, however globalization and climate change have led to an expansion of habitat for mosquitoes, increasing the risk of mosquito borne disease transmission. *Culex* spp. mosquitoes are now ubiquitous worldwide, capable of inhabiting tropical, subtropical, and temperate regions (Ciota and Kramer, 2013). They are principal vectors for a variety of arboviruses such as Japanese encephalitis virus (Sharma et al., 2021; Hernández-Triana et al., 2022), St. Louis encephalitis virus (Reisen, 2003), Usutu virus, and West Nile virus (WNV) (Brugman et al., 2018). Due to the absence of effective vaccine and treatment options for most arboviruses, including *Culex*-borne viruses, prevention of transmission at the vector level remains the most likely means of reducing disease burden. Developing a greater understanding of mosquito immune pathways may translate into novel vector control methods. However, much of our current understanding of mosquito-virus interactions comes from *Aedes aegypti*. There is a need to build and expand on these findings in *Culex* spp. mosquitoes.

The first step of any immune response is sensing the presence of a microbial infection. Innate immune responses are generally activated by pattern recognition receptors (PRRs), which recognize pathogen-associated molecular patterns (PAMPs) (Janeway and Medzhitov, 2002; Lu et al., 2020). Mosquito antiviral responses include several conserved signal transduction pathways resulting in antiviral effector gene expression and restriction of arbovirus replication. These pathways include the Janus kinase/signal transducer and activator of transcription (JAK/STAT), Toll, and immune deficiency (Imd) pathways, all of which can be activated by a variety of PAMPs (Kumar et al., 2018; Tikhe and Dimopoulos, 2021). In mammals, dsRNA is a major PAMP of viruses sensed by a variety of receptors that often result in the activation of antiviral and inflammatory pathways (Chen and Hur, 2022). In mosquitoes, the helicase and endonuclease Dicer-2 (Dcr-2) is responsible for sensing dsRNA during viral infection, but its main known function is to then process dsRNA into siRNAs that target and degrade viral genomes in a sequence-dependent manner as part of the RNA interference (RNAi) response (Bronkhorst and van Rij, 2014; Kumar et al., 2018). Aside from its very defined role in RNAi, Dcr-2 is related to mammalian Rig-I-like receptors, which sense cytosolic dsRNA and activate an interferon-based antiviral response (Deddouche et al., 2008; Baldaccini and Pfeffer, 2021). About a decade ago, researchers identified a signaling function of Dcr-2 to activate the NF-κB-like transcription factor Rel2 following WNV infection of *Culex quinquefasciatus*-derived Hsu cells (Paradkar et al., 2012; Paradkar et al., 2014). The downstream upregulation of the cytokine-like molecule Vago was shown to activate the JAK/STAT pathway (Paradkar et al., 2012). However, it was not tested whether this signaling was dsRNA mediated or initiated by another aspect of virus infection. This role in immune signaling had also been identified in *Drosophila melanogaster* Dcr-2 (Deddouche et al., 2008). Another recent study found that the synthetic dsRNA analog, poly(I:C), activated *rel2* expression in mosquito cells (Russell et al., 2021). Based on this evidence, mosquito Dcr-2 remains a strong candidate for a role in sensing viral dsRNA and eliciting a sequence-independent immune response to virus infection.

Another mechanism that could be conserved between humans and mosquitoes is dsRNA sensing via Toll receptors. The Toll pathway has been shown to be important for antiviral defenses in mosquitoes (Xi et al., 2008) and *Ae. aegypti* Toll6 (AaToll6) was recently shown to share residues important for dsRNA binding with human Toll-like receptor 3 (TLR3), a known sensor of dsRNA in humans (Angleró-Rodríguez et al., 2021). The authors also determined that numerous antimicrobial peptides (AMPs) downstream of Toll signaling were upregulated in mosquitoes cells exposed to poly(I:C) (Angleró-Rodríguez et al., 2021), which was previously used to determine that mammalian TLR3 acts as a PRR for dsRNA (Alexopoulou et al., 2001). Angleró-Rodríguez et al. also showed that extracellularly provided poly(I:C) localized to endosomes, where human TLR3 is localized and senses dsRNA. However, it remains unknown whether Toll6 also localizes to endosomes, whether it senses dsRNA and poly(I:C), and if it plays any role in antiviral responses of mosquitoes. Another open question is whether any other Toll receptors are involved in dsRNA sensing. In mammals, other TLRs besides TLR3 are also important for controlling virus infection (Lester and Li, 2014) and multiple TLRs can detect foreign nucleic acids. In *Cx. quinquefasciatus*, there are 9 Toll genes (Bartholomay et al., 2010), but none have been studied in any depth.

In this study, we aimed to elucidate if and how dsRNA sensing impacts virus replication in *Cx. quinquefasciatus*-derived Hsu cells. We used poly(I:C) to elicit an immune response and tested whether poly(I:C) priming impacts replication of the orthobunyavirus La Crosse virus (LACV). The advantage of using poly(I:C) over other long dsRNA molecules is that it will not elicit a sequence-specific antiviral response in mosquito cells. We used LACV as our model virus due to its ability to efficiently replicate in Hsu cells (Walsh et al., 2022) and its ability to be transmitted by *Culex* spp. mosquitoes (Thompson et al., 1972; Harris et al., 2015) despite being traditionally transmitted by *Aedes triseriatus* (Borucki et al., 2002). We found that LACV was reduced in poly(I:C) primed cells and further tested which AMPs are differentially regulated following poly(I:C) treatment. We used media-based treatment and transfection-based treatment in an attempt to elicit responses through endosomal and cytosolic signaling, respectively. We also found that multiple Toll receptors were upregulated following poly(I:C) treatment and, finally, determined that knockdown of *Cx. quinquefasciatus* Toll6 (CqToll6) using siRNA increased LACV replication in Hsu cells. While we were not yet able to generate conclusive evidence for any role of Toll6 in dsRNA sensing, we have shown that it is upregulated rapidly upon poly(I:C) stimulation and that Toll6 is involved in antiviral responses in *Cx. quinquefasciatus* Hsu cells.

## 2 Materials and methods

### 2.1 Cell lines

The *Cx. quinquefasciatus* ovary-derived (Hsu) cell line (Hsu et al., 1970) was grown at 27°C, and 5% CO_2_ in Dulbecco’s Modified Eagle Medium (DMEM; Corning #10-013-CV; Corning, New York, NY, USA) supplemented with 10% FBS and antibiotics (100 units mL penicillin, 100 μg/mL streptomycin, 5 μg/mL gentamicin).

### 2.2 Viruses

LACV strain R97841d was initially obtained from a human brain in Tennessee in 2012 and kindly provided by Brandy Russell, CDC Fort Collins, as a Vero p1 stock. Virus was then propagated on Vero cells and concentrated using the Amicon^®^ Ultra-15 Centrifugal Filter (Sigma-Aldrich, #UFC9010 St. Louis, MO, USA) to obtain a high titer stock. Virus titration was performed by standard plaque assay on Vero cells.

### 2.3 Poly(I:C) stimulation

Hsu cells were seeded into 24-well plates in 0.5 mL of culture medium. Cells were incubated overnight at 27°C to allow for cell adhesion. Poly(I:C) treatment was performed by replacing the culture medium with fresh medium containing 100 μg/mL (50 μg total) of high molecular weight poly(I:C) (Invivogen, tlrl-pic, San Diego, CA, USA) or the equivalent volume of physiological H_2_O. Transfection of poly(I:C) was performed using Lipofectamine RNAiMAX (Thermo Fisher, #13778075, Waltman, MA, USA) following the manufacturer’s protocol. Briefly, per well, 1.5 µL Lipofectamine RNAiMAX reagent (Thermo Fisher, #13778100, Waltman, MA, USA) was diluted in 25 µL Opti-MEM (Thermo Fisher, #31985062, Waltman, MA, USA). In parallel, 10 μg high molecular weight poly(I:C) were diluted in 25 µL Opti-MEM (Thermo Fisher, #31985062, Waltman, MA, USA). Next, the diluted poly(I:C) was added to the diluted Lipofectamine RNAiMAX reagent (Thermo Fisher, AM1626, Waltman, MA, USA), mixed by pipetting up and down, and the complex was incubated for 5 min at room temperature. An amount of 50 µL of the complex was added to cells with 450 µL fresh complete media. Cells were incubated for the indicated time periods before RNA extraction or virus infection.

### 2.4 Virus infection

Hsu were seeded into 24-well plates and infected with LACV at an MOI of 1 or 10. Briefly, per well, virus was diluted in 250 µL of DMEM (without additives) and added to each well after removal of complete culture media. For mock infected controls, 250 µL DMEM (without additives) was added to each well. Cells were incubated for 1 h at 27°C to allow for infection. After 1 h, the virus-containing media was removed, and replaced with 0.5 mL of complete culture media. The plate was then incubated at 27°C for 24 or 48 h before RNA extraction.

### 2.5 RNA extraction

RNA was extracted using the Direct-zol™ RNA miniprep kit (Zymo Research, #D4033; Irvine, CA, USA) following the manufacturer’s protocol. Briefly, culture media was removed from all wells, and 300 µL TRI reagent was added to each well. Plates were incubated with TRI reagent on a rocker for 20 min to ensure complete lysis. Cell lysates were transferred to a microcentrifuge tube and frozen at −80°C prior to RNA extraction following the provided protocol. RNA was quantified using a Qubit Flex Fluorometer (Thermo Fisher, Waltham, MA, USA).

### 2.6 cDNA synthesis and RT-qPCR

To measure AMP and Toll gene expression, cDNA was first generated from 200 ng of each RNA sample using the High-Capacity cDNA reverse transcription kit (Thermo Fisher, #4368814, Waltman, MA, USA). Quantitative real-time PCR (qRT-PCR) was then performed using iTaq Universal SYBR Green Supermix (Bio-Rad, #1725120, Hercules, CA, USA) and primers (**Table 1**) on a CFX96 Touch Real-Time PCR Detection System (Bio-Rad). Each target gene was normalized to a previously tested, reliable housekeeping gene (actin-5c) to quantify gene expression levels.

**Table 1:** Primers used for RT-qPCR of *Culex quinquefasciatus* genes and LACV RNA.

Gene	Accession Number	RT-qPCR Primers
Actin-5c	XM_038249510.1	F-CAACTGCCCAAATCGAATGAC R-CGACGCACTCTCGGAATAAA
LACV	OP962744.1	F-CAGCCCAGACAGCCATAAA R-CCCTGGTAGCATGTTGTATGT
Cec-A	XM_001861705.2	F-CTTCAACAAGCTGTTCGTCATC R-GCCAACTCCTTCCAACTTCT
Cec-B	XM_001861706.2	F-CCAACCTTCAAAACTCCTCACA R-TTGCCAAACTTCTTCAGACCAC
Def-B	XM_001862096.2	F- CAAACTCTCACGTGCACAAATC R- CACCTTCCAATTTGACACTTTCC
Def-C	XM_038251893.1	F-CTGTGACCTGTTGAGTGGATTC R-CCTTCTTGCCGTTGCAGTAT
Vago	XM_001842212.2	F-CAAATCTCTCGCTGACCTACAC R-CCCTCCAGTTCACACTTGTATT
Dicer-2	XM_038254595.1	F-GATGCAAGGGCTGGAGATAAA R-CTGCGACCTTCCTTGTAGAAC
Toll1	XM_038266672.1	F-AGCAACCACGTCAAACTACTC R-GGGTTCGAGTTCGGTGATTT
Toll4	XM_001850090.2	F-CTCACACTTACCGAGACATTCC R-CGTGCTTCGCGATTTGTTTAT
Toll6	XM_001868754.2	F-GAAGGAGAAGAACGGAACCAA R-CAACAACAGCGGCAACATATC
Toll7	XM_001866820.2	F-GGAAACTTCCTGACCGACATAA R-CGTAATCGAACCACACCAGAT
Toll8	XM_038255442.1	F-AGCAACGTGGAGGTGATTAG R-TTCAGCTCGGTTATCTGATTGT
Toll9A	XM_001848147.2	F-CACGGATGGAGAATCCCTTTAT R-CAACTTGCTGTGGGACAAATC
Toll9B	XM_038258523.1	F-GCACAACGGCCAACATTATTA R-CGTGATCCCACTTGGAGATATAG
Toll10	XM_038254632.1	F-GCTGCACATGGAGAACAATTAC R-ATCGCGTTATGGTCCAGATAAA
Toll11	XM_038252798.1	F-CTCTCGTCGCTGGTGTATTT R-CAGCTCGGAAATGTTGTTCTTG

To determine knockdown efficiency and LACV RNA levels, 100 ng of each RNA sample was used directly in a one-step qRT-PCR reaction using iTaq Universal SYBR Green One-Step Kit (Bio-Rad, #1725150) or iTaq Universal Probes One-Step Kit (Bio-Rad #1725140). *Toll6* and LACV RNA levels were again normalized to actin-5c as a housekeeping gene.

### 2.7 siRNA knockdown

Transfection of gene-specific siRNA and a non-specific siRNA control (RLuc) was done by reverse transfection using Lipofectamine RNAiMAX (Thermo Fisher, #13778075, Waltman, MA, USA) following the manufacturer’s protocol. Briefly, per well, 3 µL of 5 µM siRNA was diluted with 1.5 µL Lipofectamine RNAiMAX reagent (Thermo Fisher, #13778100, Waltman, MA, USA) in Opti-MEM (Thermo Fisher, #31985062, Waltman, MA, USA) to a final volume of 100 μL. The solution was mixed by pipetting up and down, incubated for 20 min at room temperature, and then each complex was added to its respective well in a 24-well plate. Hsu cells in 0.5 mL of culture medium were then added to each well. Cells were incubated for 48 h before RNA extraction or virus infection.

### 2.8 Phylogenetic analysis

*Cx. quinquefasciatus*, *Ae. aegypti, A. gambiae*, *D*. *melanogaster*, and *H. sapiens* Toll receptor protein sequences were collected from the VectorBase and NCBI databases (**Table 2**). *Culex tarsalis* protein sequences were identified from the published *Cx. tarsalis* genome (Main et al., 2021) using blastp in the BLAST command line to search for Toll receptors with the closely related *Cx*. *quinquefasciatus* Toll protein sequences. All *Cx. tarsalis* Toll protein sequences were confirmed to contain a Toll/interleukin-1 receptor (TIR) domain and leucine rich (LRR) motifs using InterPro (Paysan-Lafosse et al., 2023). Sequence alignment and tree generation was performed as done previously (Zhang et al., 2021) with minor variations. Briefly, Toll sequences from all species were aligned using MAFFT (v7.520) with the E-INS-I alignment algorithm to account for sequences with multiple conserved domains and long gaps. The phylogenetic tree was generated using the ExaBayes (version 1.4.1) tool with the optimal amino acid substitution model for the data chosen by default. The analysis was initiated with a random seed and ran for 1,000,000 generations, sampling every 500 generations. Visualization and annotation were done in FigTree (version 1.4.4).

**Table 2:** The protein accession numbers of TLR and Toll sequences used in phylogenetic analysis.

Species	Gene	Accession Number
*Homo sapiens*	TLR1	AAY85640.1
*Homo sapiens*	TLR2	AAC34133.1
*Homo sapiens*	TLR3	ABC86908.1
*Homo sapiens*	TLR4	AAY82270.1
*Homo sapiens*	TLR5	AAZ17468.1
*Homo sapiens*	TLR6	ABW37063.1
*Homo sapiens*	TLR7	AAZ99026.1
*Homo sapiens*	TLR8	AAQ88663.1
*Homo sapiens*	TLR9	AAZ95519.1
*Homo sapiens*	TLR10	AAY78485.1
*Aedes aegypti*	Toll1A	XP_021709288.1
*Aedes aegypti*	Toll1B	XP_021694310.1
*Aedes aegypti*	Toll4	XP_021713350.1
*Aedes aegypti*	Toll5A	XP_021703638.1
*Aedes aegypti*	Toll5B	XP_021708988.1
*Aedes aegypti*	Toll6	XP_021710322.1
*Aedes aegypti*	Toll7	XP_001655730
*Aedes aegypti*	Toll8	XP_001649813
*Aedes aegypti*	Toll9A	XP_021698584
*Aedes aegypti*	Toll9B	XP_021704623
*Aedes aegypti*	Toll10	XP_001648238.1
*Aedes aegypti*	Toll11	XP_021704787.1
*Culex quinquefasciatus*	Toll1	XP_038122600.1
*Culex quinquefasciatus*	Toll4	XP_001850142.1
*Culex quinquefasciatus*	Toll6	XP_038107429.1
*Culex quinquefasciatus*	Toll7	XP_001866855.2
*Culex quinquefasciatus*	Toll8	XP_038111370.1
*Culex quinquefasciatus*	Toll9A	XP_001848199.2
*Culex quinquefasciatus*	Toll9B	XP_038114451.1
*Culex quinquefasciatus*	Toll10	XP_038110560.1
*Culex quinquefasciatus*	Toll11	XP_038108726.1
*Culex tarsalis*	Toll1	*
*Culex tarsalis*	Toll4	*
*Culex tarsalis*	Toll6	*
*Culex tarsalis*	Toll7	*
*Culex tarsalis*	Toll8	*
*Culex tarsalis*	Toll9	*
*Culex tarsalis*	Toll10	*
*Culex tarsalis*	Toll11	*
*Anopheles gambiae*	Toll1A	XP_309197.1
*Anopheles gambiae*	Toll1B	XP_311355.3
*Anopheles gambiae*	Toll5A	XP_560220.3
*Anopheles gambiae*	Toll5B	XP_311384.3
*Anopheles gambiae*	Toll6	XP_320172.2
*Anopheles gambiae*	Toll7	XP_320221.4
*Anopheles gambiae*	Toll8	XP_551799.2
*Anopheles gambiae*	Toll9	XP_565221.2
*Anopheles gambiae*	Toll10	XP_309458.4
*Anopheles gambiae*	Toll11	XP_309461.4
*Drosophila melanogaster*	Toll-1	NP_001262995.1
*Drosophila melanogaster*	Toll-2	NP_476814.1
*Drosophila melanogaster*	Toll-3	NP_649719.2
*Drosophila melanogaster*	Toll-4	NP_523519.2
*Drosophila melanogaster*	Toll-5	NP_001285901.1
*Drosophila melanogaster*	Toll-6	NP_001246766.1
*Drosophila melanogaster*	Toll-7	NP_523797.1
*Drosophila melanogaster*	Toll-8	NP_524757.1
*Drosophila melanogaster*	Toll-9	NP_001246846.1

*Culex tarsalis Toll protein sequences are provided in **Supplementary Material 1**.

### 2.9 Statistical analysis

Statistical analysis was performed using ANOVA or unpaired t-test in GraphPad Prism as indicated in figure legends. Outliers from select individual experiments were excluded from the data using the Grubb’s test with a significance level of 0.05 in GraphPad Prism.

## 3 Results

### 3.1 Poly(I:C) priming reduces LACV replication in *Cx. quinquefasciatus* cells

**Figure 1:**
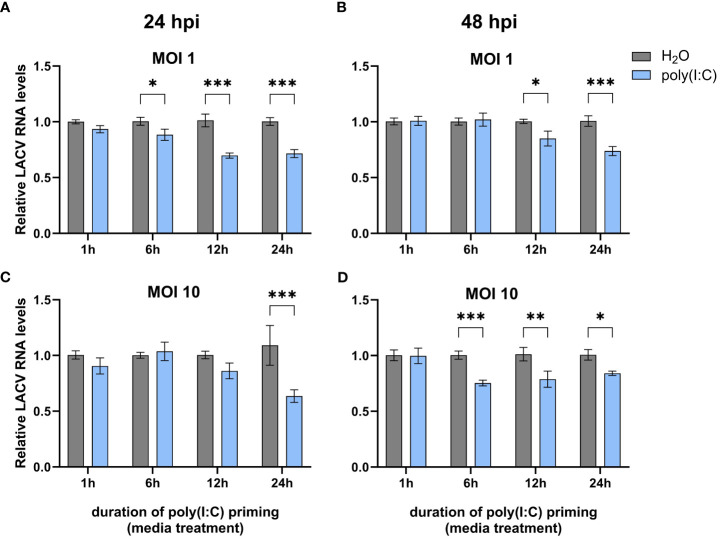
Effect of poly(I:C) priming on LACV RNA levels. Hsu cells were treated with poly(I:C) for 1, 6, 12, or 24 h prior to infection with LACV at MOI 1 **(A, B)** or 10 **(C, D)**. Cellular RNA was extracted and LACV RNA levels were measured by RT-qPCR at 24 hpi **(A, C)** or 48 hpi **(B, D)** and normalized to actin-5c (housekeeping gene). Bars represent the mean +/- SEM of 2 independent experiments with 4 biological replicates each. Significant changes in viral RNA abundance compared to the H_2_O control are shown as * p < 0.05, ** p < 0.01, *** p < 0.001 as analyzed by two-way ANOVA.

In order to determine if poly(I:C) elicits an antiviral immune response in *Cx. quinquefasciatus*, we primed Hsu cells with 100 μg mL of poly(I:C) or water prior to infection with LACV. As the duration of poly(I:C) treatment can affect the expression of cytokines in mammals (Kato et al., 2006) and immune effector genes in *Ae. aegypti* (Angleró-Rodríguez et al., 2021), we chose four different timepoints of poly(I:C) priming time. Hsu cells primed with poly(I:C) for 6, 12, and 24 hours led to a modest but significant (p < 0.05) decrease in LACV RNA levels 24 hours post infection (hpi) at an MOI of 1 (**Figure 1A**). Poly(I:C) priming for 12 and 24 h also resulted in a significant decrease of LACV RNA at 48 hpi (**Figure 1B**). To see if a similar effect was maintained at a higher viral load, we primed Hsu cells and infected at an MOI of 10. At 24 hpi, only 24 hours of priming had been able to reduce LACV RNA levels (**Figure 1C**). However, by 48 hpi LACV RNA levels were again significantly reduced in cells primed with poly(I:C) for 6, 12, and 24 h prior to infection (**Figure 1D**). Overall, these results showed that poly(I:C) treatment prior to virus infection can reduce LACV RNA levels in Hsu cells across different MOIs and infection periods.

### 3.2 Poly(I:C) treatment induces modest AMP upregulation in *Cx. quinquefasciatus* cells

**Figure 2:**
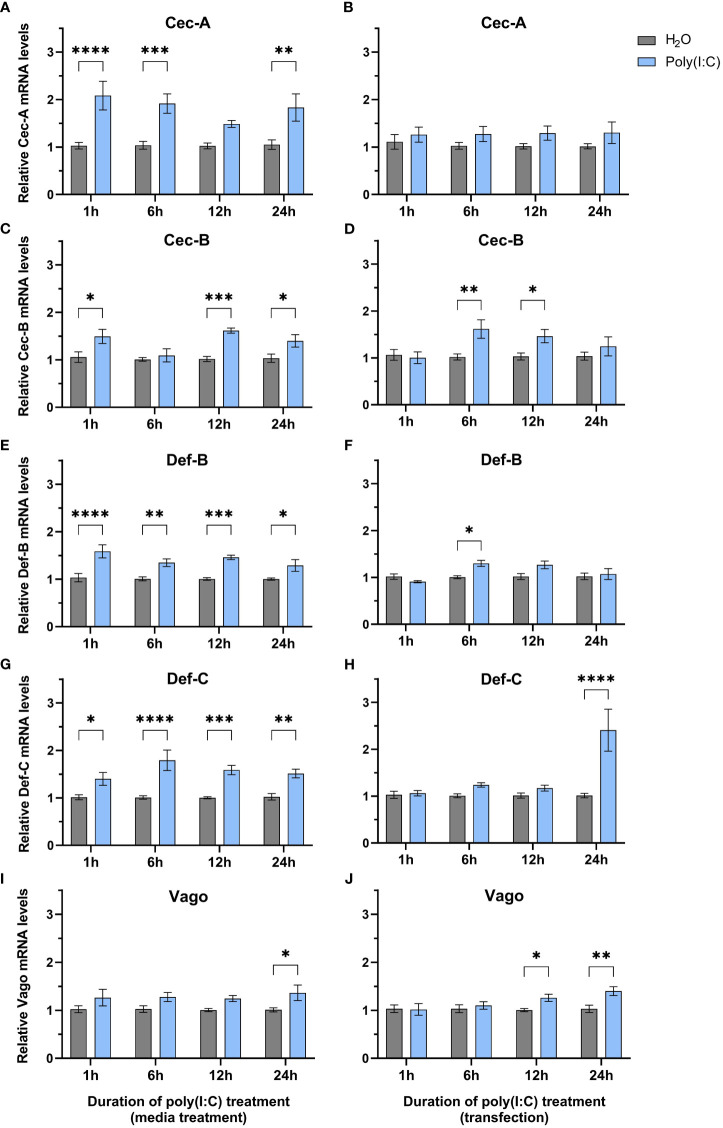
Expression of select immune genes after treatment with poly(I:C). Hsu cells were treated with poly(I:C) alone **(A, C, E, G, I)** or with poly(I:C) plus a transfection reagent **(B, D, F, H, J)** for 1, 6, 12, or 24 h. Cellular RNA was extracted and gene expression was measured by RT-qPCR and normalized to actin-5c (housekeeping gene). Bars represent the mean +/- SEM of 3 independent experiments with 4 biological replicates each. Significant changes in mRNA abundance compared to the H_2_O control are shown as * p < 0.05, ** p < 0.01, *** p < 0.001, **** p < 0.0001, as analyzed by two-way ANOVA.

Since we observed a significant decrease in LACV replication, we wanted to determine if any immune genes were differentially regulated following poly(I:C) treatment. We delivered poly(I:C) to Hsu cells with or without a transfection reagent to mimic cytosolic dsRNA sensing and extracellular sensing, respectively, and measured immune effector gene expression at different timepoints post stimulation by RT-qPCR. We measured the mRNA levels of cecropins, defensins, and the cytokine-like molecule Vago, all of which showed increased mRNA levels at varying durations of poly(I:C) treatment with and/or without a transfection reagent (**Figures 2A–H**). Cecropin-A (Cec-A) mRNA levels were significantly (p < 0.05) increased at 1, 6, and 24 h post treatment (**Figure 2A**), but there was no increase in Cec-A mRNA following poly(I:C) transfection (**Figure 2B**). Another cecropin, Cec-B, was upregulated at 1, 6, and 24 h post treatment without a transfection reagent (**Figure 2C**). Additionally, transfected poly(I:C) upregulated Cec-B’s mRNA levels at 6 and 12 h, returning to basal levels at 24 h (**Figure 2D**). Transcript abundance of Defensin-B (Def-B) was increased at all timepoints after treatment without a transfection reagent (**Figure 2E**), with only a slight uptick at 6 h post poly(I:C) transfection (**Figure 2F**). Def-C mRNA was also upregulated at all timepoints when poly(I:C) was added alone (**Figure 2G**), but only after 24 h of transfected poly(I:C) treatment (**Figure 2H**).

In contrast to studies in *Ae. aegypti* Aag2 cells (Angleró-Rodríguez et al., 2021; Russell et al., 2021), we found that *vago* is upregulated after poly(I:C) treatment without (**Figure 2I**) and with a transfection reagent (**Figure 2J**). While statistically significant (p < 0.05), this increase in *vago* expression was only modest with a 1.4-fold increase at 24 h post treatment (**Figure 2I**) and a 1.3-fold and 1.4-fold increase at 12 and 24 h post transfection, respectively (**Figure 2J**).

### 3.3 CqToll6 is involved in antiviral defenses

**Figure 3:**
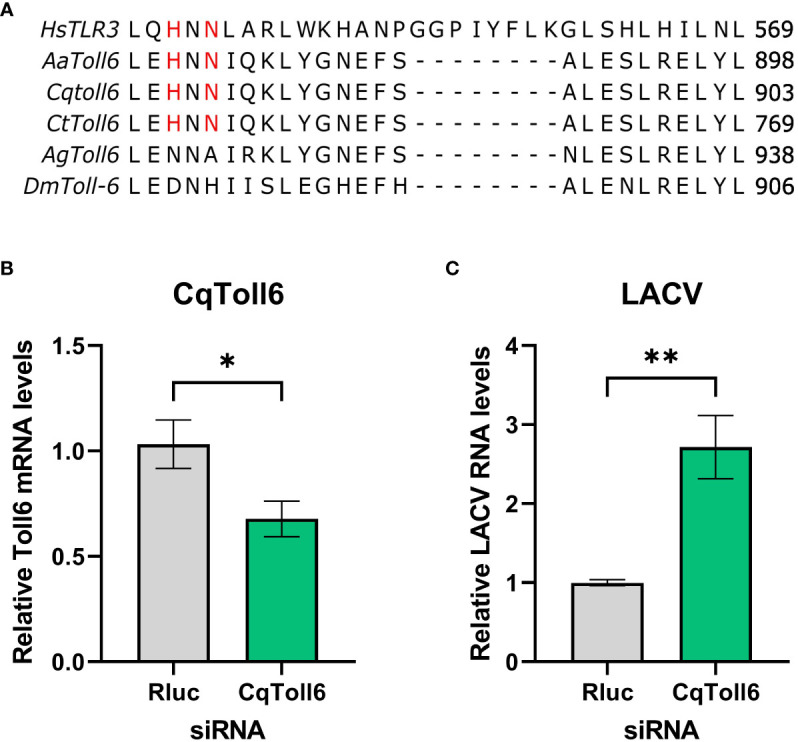
CqToll6 knockdown increases LACV replication. The protein sequences of Toll6 from *Culex quinquefasciatus* (Cq), *Culex tarsalis* (Ct), *Aedes aegypti* (Aa), *Anopheles gambiae* (Ag), and *Drosophila melanogaster* (Dm) and TLR3 from *Homo sapiens* (Hs) were aligned using Clustal Omega **(A)**. The histidine and asparagine residues important for TLR3 dsRNA binding are shown in red. CqToll6 was silenced in Hsu cells using siRNA and validated 48 hours post siRNA transfection **(B)**. Hsu cells were then infected with LACV MOI 1 **(C)**. Cellular RNA was extracted at the time of infection **(B)** or at 48 hpi **(C)**. CqToll6 knockdown efficiency **(B)** and viral RNA levels **(C)** were determined by RT-qPCR and normalized to actin-5c (housekeeping gene). Bars are the mean +/SEM of 2 independent experiments with 4 biological replicates each. Significant changes in RNA abundance compared to the *Renilla luciferase* targeting control are shown as * p < 0.05, ** p < 0.01 as analyzed by unpaired t-test.

In searching for a potential receptor responsible for dsRNA sensing in mosquitoes, Toll6 in *A. aegypti* was recently shown to share a conserved amino acid motif with human TLR3 that is required for dsRNA recognition (Angleró-Rodríguez et al., 2021). However, little is known about other mosquito *toll6* genes. Protein sequence alignment showed that these amino acid residues are also conserved in *Cx. quinquefasciatus* and *Culex tarsalis*, but not in *D. melanogaster* or *Anopheles gambiae*) (**Figure 3A**). We thus hypothesize that CqToll6 is involved in dsRNA sensing.

We first wanted to investigate whether CqToll6 is involved in controlling virus replication in Hsu cells. We used siRNA-mediated knockdown of CqToll6 expression in Hsu cells and infected with LACV at an MOI 1. While statistically significant, CqToll6 RNA levels were only reduced modestly (**Figure 3B**). However, following knockdown, we infected cells with LACV and, at 48 hpi, there was a significant increase in LACV cellular RNA levels (**Figure 3C**). These results indicate that CqToll6 is antiviral against LACV even after a modest knockdown. Due to the difficulty of obtaining a reliable knockdown in CqToll6 expression, we were unable to directly investigate its role in poly(I:C) sensing.

### 3.4 Mosquito Toll phylogeny

**Figure 4:**
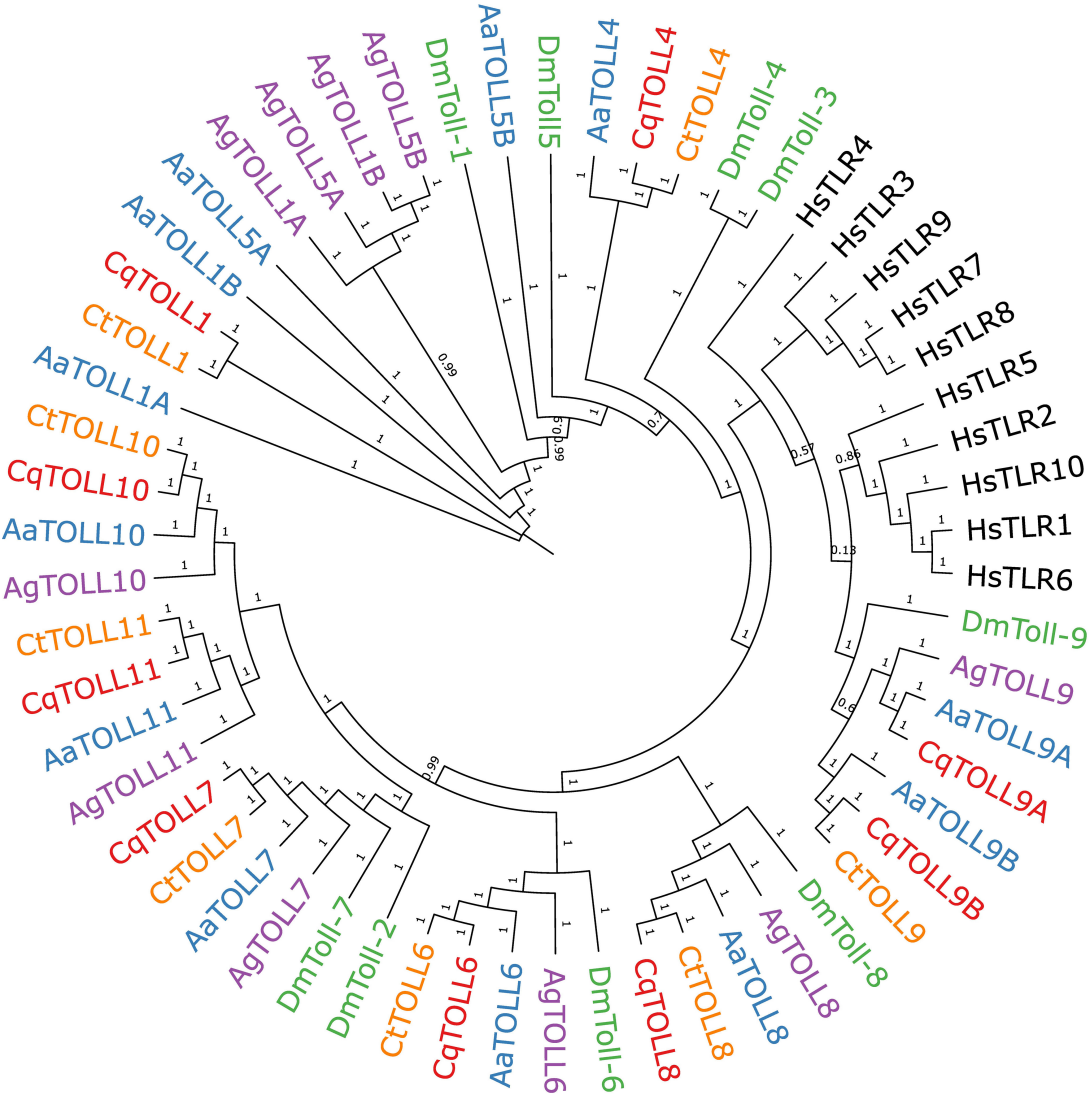
Bayesian inference of phylogeny of *Culex quinquefasciatus* (Cq), *Culex tarsalis* (Ct), *Aedes aegypti* (Aa), *Anopheles gambiae* (Ag), *Drosophila melanogaster* (Dm), and *Homo sapiens* (Hs) full length Toll receptor protein sequences. Branch nodes are labeled with posterior probabilities.

We also wanted to extend our understanding of the Toll receptor repertoire in *Culex* species mosquitoes. It is now known that receptors besides TLR3, such as TLR10, can sense poly(I:C) in human cells (Lee et al., 2018), suggesting that multiple mosquito Toll receptors may be responsible for sensing poly(I:C). There are nine Toll genes in *Cx. quinquefasciatus* mosquitoes, all of which can be detected in the head, thorax, and abdomen of adult *Cx. quinquefasciatus* mosquitoes (**Supplemental Figure 1**). To build a comprehensive phylogeny of Toll receptors in vector mosquitoes, we first set out to identify all Toll receptors in the recently assembled genome of *Cx. tarsalis* (Main et al., 2021). We used the protein sequences of *Cx. quinquefasciatus* Toll genes to BLAST for homologous genes in the *Cx. tarsalis* genome. In our final phylogenetic analysis, we included protein sequences of Toll genes from four mosquito species, *D. melanogaster* and TLRs from humans to generate a phylogenetic tree (**Figure 4**). Our findings show a clear subcluster of Toll1/5 in mosquitoes that has been described previously (Christophides et al., 2002; Kanzok et al., 2004). However, both *Culex* spp. appear to have lost the Toll5 gene and do not contain the Toll1 gene duplication seen in *Ae. aegypti* and *An. gambiae.* Both *Culex* spp. also possess a Toll4 that belongs to the larger Toll1/3/4/5 family. We found that Toll9 clustered closely with all human TLRs, suggesting Toll9 may be a conserved PRR considering that mammalian TLRs are essential players in recognizing infections (Leulier and Lemaitre, 2008; Lester and Li, 2014). We could only find one copy of *Cx. tarsalis* Toll9, despite the presence of two Toll9 copies in *Cx. quinquefasciatus* and *Ae. aegypti*. Mosquito Tolls 6-8 tended to cluster with their respective *D. melanogaster* ortholog in our analysis. The mosquito-specific Toll10 and 11 were conserved between the four mosquito species in our analysis.

### 3.5 Select *toll* genes are differentially regulated following poly(I:C) exposure and LACV infection

**Figure 5:**
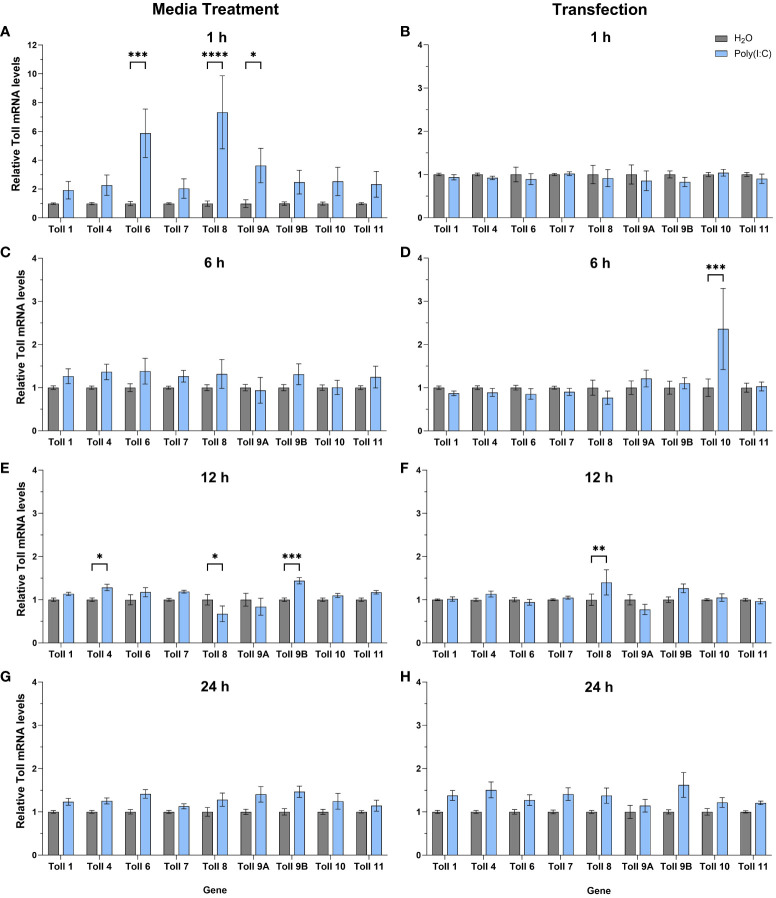
Expression of *toll* genes after treatment with poly(I:C). Hsu cells were treated with poly(I:C) alone **(A, C, E, G)** or with poly(I:C) plus a transfection reagent **(B, D, F, H)**, for 1, 6, 12, or 24 hours. Cellular RNA was extracted and gene expression was measured by RT-qPCR and normalized to actin-5c (housekeeping gene). Bars represent the mean +/- SEM of 3 independent experiments with 4 biological replicates each. Significant changes in RNA abundance compared to the H_2_O control are shown as * p < 0.05, ** p < 0.01, *** p < 0.001, **** p < 0.0001 as analyzed by two-way ANOVA.

To help further our functional understanding of all *Culex* Toll receptors, we treated Hsu cells with poly(I:C) with or without a transfection reagent and measured mRNA levels of all nine *toll* genes at 1, 6, 12 and 24 h (**Figures 5A–H**). In cells treated with poly(I:C) alone, *toll6*, *8*, and *9A* were significantly upregulated after 1 h of treatment (**Figure 5A**) showing the largest increase in *toll* expression of all treatments and timepoints. In contrast, no *toll* genes were differentially regulated following poly(I:C) transfection for 1 h (**Figure 5B**). By 6 h post media treatment, all *toll* genes were at comparable levels in poly(I:C) and water treated cells (**Figure 5C**). However, following 6 h of transfection with poly(I:C), *toll10* was significantly upregulated (**Figure 5D**). After 12 h of treatment, media treatment of poly(I:C) resulted in the upregulation of *toll9b* and *toll4* (**Figure 5E**). However, *toll8* was slightly downregulated 12 h post media treatment (**Figure 5E**), while it was upregulated 12 h post transfection (**Figure 5F**), possibly suggesting a difference in the timing of signaling effects. By 24 h of treatment, none of the *toll* genes showed significant changes in expression, despite a small trend for upregulation of multiple *toll* genes (**Figures 5G, H**).

**Figure 6:**
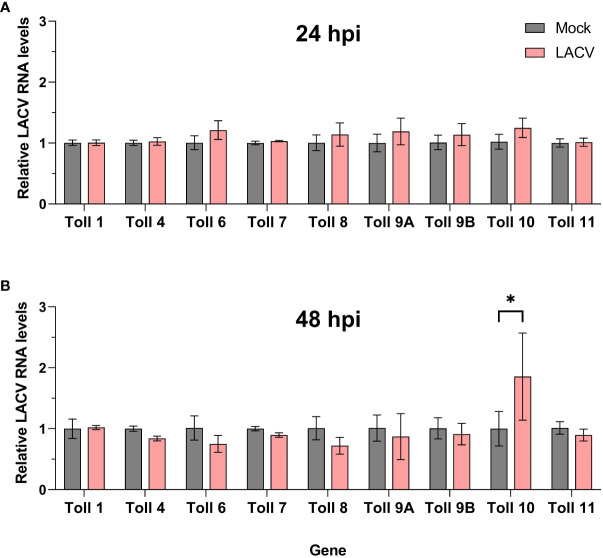
Expression of *toll* genes after infection with LACV. Hsu cells were infected with LACV MOI 1 for 24 **(A)** or 48 **(B)** hours. Cellular RNA was extracted and LACV RNA was measured by RT-qPCR and normalized to actin-5c (housekeeping gene). Bars represent the mean +/- SEM of 2 independent experiments with 4 biological replicates each. Significant changes in RNA abundance compared to the mock infected control are shown as * p < 0.05.

We then sought to determine the expression profile of *Cx. quinquefasciatus toll* genes in Hsu cells after infection with LACV. At 24 hpi, no *toll* genes were differentially regulated in Hsu cells compared to mock-infected cells (**Figure 6A**). However, by 48 hpi, there was a modest, but significant upregulation of *toll10* expression in LACV infected cells (**Figure 6B**). No other *toll* genes were differentially regulated at 48 hpi with LACV.

## 4 Discussion

In this study, we sought to build on the knowledge of how mosquitoes sense virus infection to mount an immune response. In particular, we were interested in the response of *Cx. quinquefasciatus* to the dsRNA mimic poly(I:C). We found that poly(I:C) treatment elicits an antiviral immune response in *Cx. quinquefasciatus* Hsu cells that limits LACV replication. This is consistent with studies in multiple cell lines of human origin that have shown varying treatment times with poly(I:C) can limit LACV titers (Luby, 1975; Monteiro et al., 2019). The impact on LACV replication in our study was modest and highly dependent on the duration of poly(I:C) priming prior to infection, with longer treatments more consistently reducing viral RNA levels. This finding differed from studies performed in human cells with CHIKV (Li et al., 2012) and mosquito cells with DENV (Angleró-Rodríguez et al., 2021) where 1 h of poly(I:C) priming was sufficient to reduce virus replication. However, we did observe the upregulation of select immune effector genes after only 1 h of poly(I:C) treatment. It is possible that other genes, ones not measured here, are responsible for controlling LACV replication in Hsu cells and that these are only upregulated after prolonged poly(I:C) incubation. It is worth noting that Hsu cells are slow growing cells compared to, for example, *Ae. aegypti* Aag2 cells, which might impact the exact timing of transcriptional changes compared to other mosquito cell lines. Transcriptomic profiling of poly(I:C) treated Hsu cells and *Cx. quinquefasciatus* mosquitoes at varying timepoints would help identify additional factors that may be involved in an antiviral response. It has also been shown that priming Aag2 cells with Rift Valley fever virus results in higher AMP expression after bacterial challenge than virus infection or bacterial challenge alone (Laureti et al., 2023). It may thus be worthwhile to measure AMP expression after poly(I:C) treatment and virus infection to determine if a similar priming effect is observed when a viral PAMP is used prior to a virus infection.

Our finding that certain Cecropins and Defensins are upregulated after poly(I:C) treatment in *Cx. quinquefasciatus* Hsu cells indicates a conserved response across mosquito species. This result builds on similar findings from two recent studies in *Ae. aegypti* Aag2 cells (Angleró-Rodríguez et al., 2021; Russell et al., 2021). One AMP, AaCec-E, was upregulated in both studies and expression of its *Cx. quinquefasciatus* ortholog, Cec-A, was also induced in Hsu cells here. However, Cec-A was not upregulated in Hsu cells when poly(I:C) was added with a transfection reagent, while Russell et al. used transfected poly(I:C) when measuring AaCec-E expression (Russell et al., 2021). This discrepancy could be due to inherent differences in protocols or could point towards a biological difference in the receptors that are responsible for sensing intracellular poly(I:C) when it is transfected. Poly(I:C) delivered to cells without a transfection reagent predominantly localizes to endosomes in many cell types, including mosquito cells (Angleró-Rodríguez et al., 2021), or activates receptors on the cell surface (Palchetti et al., 2015). A transfection reagent allows poly(I:C) to localize to the cytosol (Palchetti et al., 2015), likely initiating cytosolic signaling responses separate from those of the endocytic pathways triggered by simple addition to the media. Yet, in some human cell types, transfected poly(I:C) may be delivered to endosomes (Matsumoto and Seya, 2008) and may thus still be sensed there as well. Given the variability in poly(I:C) localization and the fact that different receptors are likely responsible for detecting poly(I:C) in those compartments, we feel it is important to highlight when a transfection reagent is used in experiments aiming to study potential dsRNA receptors. It will ultimately be crucial to determine which, if any, of the AMPs studied here have a direct antiviral role and what pathways regulate them.

The cytokine Vago was initially shown to be responsible for controlling DCV infection in flies (Deddouche et al., 2008). Later, Vago was found to be upregulated in response to WNV infection and to act as a cytokine-like molecule that activated the JAK-STAT pathway in *Cx. quinquefasciatus* Hsu cells (Paradkar et al., 2012; Paradkar et al., 2014). Our finding that *vago* mRNA is modestly but significantly (p < 0.05) upregulated in Hsu cells after treatment with poly(I:C) with and without a transfection reagent provides some evidence that dsRNA can act as a viral stimulus that induces *vago* expression in Hsu cells. Given that *vago* upregulation in response to virus infection is dependent on Dcr-2 (Deddouche et al., 2008; Paradkar et al., 2012; Paradkar et al., 2014), which is capable of sensing dsRNA (Blair and Olson, 2014), it follows that poly(I:C) might stimulate *vago *via Dcr-2 sensing. However, two recent studies have found *vago* to be unresponsive to poly(I:C) in *Ae. aegypti* Aag2 cells (Angleró-Rodríguez et al., 2021; Russell et al., 2021). Russell et al. recall that *Ae. aegypti* encodes two *vago* genes, and that they could only detect the expression of *vago2* without a notable increase, while Angleró-Rodríguez et al. measured *vago1* in Aag2 cells and observed no significant increase following poly(I:C) treatment (Asad et al., 2018; Angleró-Rodríguez et al., 2021; Russell et al., 2021). We, along with Paradkar et al., measured the expression of *vago1* in Hsu cells (Paradkar et al., 2012). Given that *vago* is upregulated *in vivo* in *Ae. aegypti* in response to YFV (Colpitts et al., 2011), we speculate that different experimental conditions or cell line specific effects may impact *vago* expression in Aag2 cells. However, there likely remains some variability in the response of *vago* depending on the specific virus-vector pairing.

As TLR3 is well-known to sense dsRNA (Alexopoulou et al., 2001), and AaToll6 was recently shown to share key residues important for dsRNA binding (Bell et al., 2006; Angleró-Rodríguez et al., 2021), we wanted to determine if other mosquito Toll6 sequences also shared these conserved residues. When we aligned TLR3 with Toll6 of four mosquito species and *D. melanogaster*, only *Culex* spp. and *Aedes* spp., which are important viral vectors, had these key residues conserved in Toll6. This may indicate the importance of Toll6 for sensing viral infection in these species. Given that TLR3 has been shown to recognize dsRNA and induce a type I IFN-mediated response, it is no surprise that it serves an antiviral role against a wide array of viruses in humans (Chen et al., 2021). For example, multiple studies have found TLR3 crucial for controlling dengue virus replication in human cells (Tsai et al., 2009; Nasirudeen et al., 2011). Therefore, we investigated if Toll6 is antiviral in *Cx. quinquefasciatus* Hsu cells. After infection with LACV for 48 h, there was a significant increase in viral RNA levels, indicating that CqToll6 serves an antiviral role in Hsu cells. Additional studies are warranted to determine whether this antiviral role is mediated by direct dsRNA binding or other mechanisms, and to understand the broader impact it has on arboviruses in mosquitoes. Since siRNA and dsRNA knockdown of Toll6 is difficult to achieve, based on our experience, and introduces a confounding dsRNA molecule to the cells, generating a CRISPR/Cas9 knockout cell line and a CqToll6 expression plasmid would be advantageous for future studies.

We also wanted to build on the phylogenetic understanding of Tolls in mosquito species, given how important TLRs are in human innate immune responses to virus infections (Lester and Li, 2014) and how little is known of mosquito Toll receptors. In addition, in the context of poly(I:C), it is now known that receptors besides TLR3 can sense poly(I:C) in human cells, such as TLR10 (Lee et al., 2018). It is thus plausible to hypothesize that other Toll receptors, besides Toll6, may be responsible for poly(I:C) sensing in mosquitoes. While we used a similar approach to Zhang et al. in our analysis, we observed differences in Toll clustering (Zhang et al., 2021). In that study, the authors only included a subset of mosquito Toll protein sequences in their analysis and found that invertebrate Toll9s clustered with TLR4 (Zhang et al., 2021). Instead, we included the full-length protein sequences for all Tolls in the species we focused on and were able to add to previous studies, finding that mosquito Toll9s, including *Culex* spp., clustered with another set of human TLRs (Imler and Zheng, 2004; Kanzok et al., 2004; Waterhouse et al., 2007). Mammalian TLRs, in general, are considered *bona fide* PRRs. The clustering of insect Toll9s with human TLRs has been proposed to be due to the number of cysteine clusters present on the ectodomains. All mammalian TLRs harbor a single cysteine cluster, while most insect Tolls, besides Toll9, possess multiple cysteine clusters (Imler and Zheng, 2004; Leulier and Lemaitre, 2008; Brennan and Gilmore, 2018). Additionally, only the intracellular TIR domain of insect Toll9 clusters with those of human TLRs, and, based on this clustering, it has been proposed that most insect Tolls likely evolved separately, and their function in immunity has been questioned (Imler and Zheng, 2004). While Toll9 has been shown to be involved in *Drosophila* immunity (Ooi et al., 2002), no Toll9 receptor has been studied in mosquitoes to our knowledge. It is also worth noting that even between *Diptera* species, the function of any particular Toll receptor may not be conserved (Lima et al., 2021). However, given the demonstrated importance of the Toll pathway in mosquitoes (Xi et al., 2008), the implication of other Toll genes involved in *Drosophila* antiviral immunity (Nakamoto et al., 2012), and the effect of CqToll6 knockdown seen here, it is reasonable to think that other mosquito Toll receptors also play a role in immune defense against viruses. It will be important to determine the downstream effects any individual Toll receptors may have in response to viral infection, as it is largely unknown which Tolls feed into the canonical mosquito Toll signaling pathway. It was recently shown that the cytokine-like molecule Spaetzle binds AaToll5a and leads to AMP upregulation (Saucereau et al., 2022). Whether transcription such as this results from any Toll receptor sensing a viral PAMP directly remains to be seen.

It has been shown that AaToll6 is upregulated after poly(I:C) treatment in Aag2 cells (Angleró-Rodríguez et al., 2021). In human cells, poly(I:C) induces the upregulation of TLR3 (Bsibsi et al., 2006; Huang et al., 2006), but also other TLRs, such as TLR2 (Melkamu et al., 2009) and TLR4 (Anabel et al., 2014). In addition to poly(I:C) treatment, it has also been shown in human cells and patients that RNA viruses, including Zika virus, can upregulate TLR3 in a positive-feedback fashion (Tanabe et al., 2003; da Silva et al., 2019). Here, we found that the most upregulation of *toll* genes was in response to non-transfected poly(I:C) after 1 h of treatment. These included *toll6*, which complements Angleró-Rodríguez et al. studies in Aag2 cells (Angleró-Rodríguez et al., 2021), and *toll8* and *toll9A* as well. However, *toll6* was not upregulated after LACV infection, which is not reminiscent of TLR3 in multiple studies in human cells (Tanabe et al., 2003; da Silva et al., 2019). It is possible that *toll6* upregulation occurred at other timepoints post infection that we did not investigate, in particular earlier timepoints post infection. The expression of *toll-8* in *Drosophila* was shown to be upregulated after Sindbis virus infection (Xu et al., 2012). In our study, only poly(I:C), and not LACV infection, triggered the upregulation of *toll8* in Hsu cells. This may again be a result of timing or indicate that LACV can antagonize dsRNA sensing and signaling. The fact that *toll9A*, and to some extent *toll9B*, were upregulated after poly(I:C) treatment encourages future studies into these genes. The Toll receptor that stands out as being the most upregulated after transfected poly(I:C) treatment was *toll10*, which was also the only upregulated *toll* gene after LACV infection in our study. It is important to note that the lack of upregulation of any particular toll receptor does not necessarily indicate a lack of potential signaling through that receptor. Likewise, it is still unclear if any observed *toll* gene upregulation is the result of positive feedback or the result of independent upstream signaling. In addition, the timing of poly(I:C) induced upregulation of *toll* genes may not directly correlate with the timing of antiviral poly(I:C) priming. Toll protein stability and turnover in Hsu cells is unknown and short increases in mRNA levels may have longer lasting impacts on Toll protein levels and activity.

One limitation of our study is that we only tested one arbovirus. Other viruses, such as WNV or Usutu virus are relevant to the infection of *Culex* spp. mosquitoes, and we aim to test the impact of poly(I:C) priming on these viruses in the future. Similarly, studies in mosquitoes *in vivo* will be required to fully understand the relevance of these dsRNA sensing mechanisms. We found that poly(I:C) stimulation results could be variable between experiments and Toll knockdown experiments were difficult to achieve. In the future, we hope to establish Toll6 knockout cell lines to further study its role during virus replication. Key experiments will require large scale transcriptomic analysis to identify other genes that are upregulated following poly(I:C) treatment.

Overall, our data indicates that poly(I:C) induces an antiviral state in *Cx. quinquefasciatus* Hsu cells, which correlated with an increase in AMP gene expression, similar to what is found in Aag2 cells. We showed that a putative dsRNA receptor in *Cx. quinquefasciatus* mosquitoes, Toll6, is antiviral against LACV. We were able to expand and update the phylogeny of Toll receptors in multiple mosquito species and analyzed the expression of all *toll* genes in response to poly(I:C) and virus infection in *Cx. quinquefasciatus* cells. While many of our results indicate only modest changes in gene expression and virus replication, this may be a result of our slow-growing cell culture system or the redundancy of multiple immune pathways. However, our work presented here will guide future efforts to uncover the exact role of specific mosquito Toll receptors in the sensing of virus infection and guide *in vivo* studies into the role of Toll receptors in mosquito antiviral immunity.

## Data availability statement

The original contributions presented in the study are included in the article/**Supplementary Material**. Further inquiries can be directed to the corresponding author.

## Ethics statement

Ethical approval was not required for the studies on animals in accordance with the local legislation and institutional requirements because only commercially available established cell lines were used.

## Author contributions

BP performed primary data collection, experimental optimization, data analysis, and manuscript drafting. KC performed Toll expression analysis as shown in **Figures 5**, **6** and helped edit the manuscript. CR conceived and supervised the project, assisted with data visualization, as well as manuscript drafting and editing. All authors have reviewed, edited, and agreed to the final version of this manuscript.
