# Supplementary material for: Elucidating the role of dsRNA sensing and Toll6 in antiviral responses of Culex quinquefasciatus cells
Source: Front Cell Infect Microbiol. 2023 Aug 30;13:1251204. doi: 10.3389/fcimb.2023.1251204 (PMC10499357; doi:10.3389/fcimb.2023.1251204)
Supplement: Supplementary file 1 [file DataSheet_1.docx]

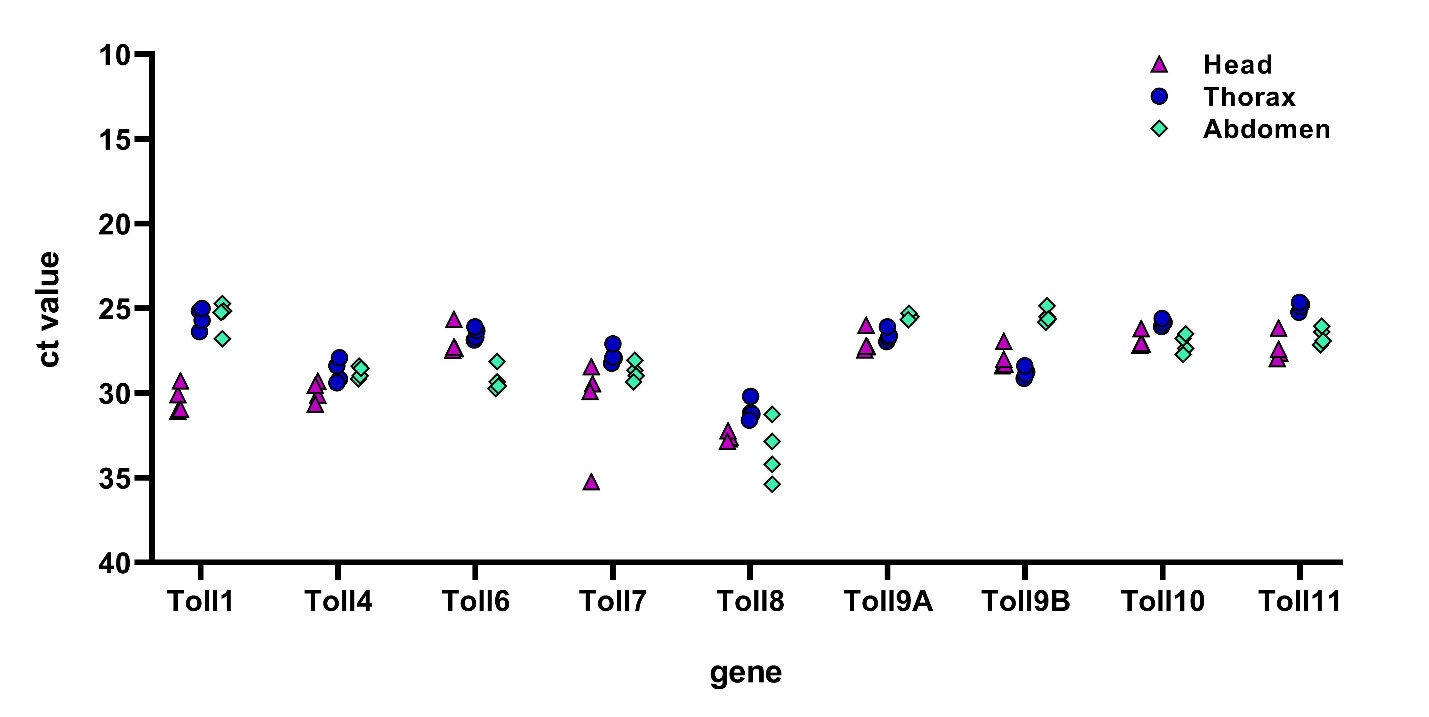
**Supplementary Figure 1.** Expression of *toll* genes in 7-day old adult *Culex quinquefasciatus* mosquitos. Four pools of five mosquito heads, thoraces, and abdomens were dissected, lysed in TRI reagent, and homogenized. RNA was extracted and 100ng from each pool was used as input for cDNA synthesis and gene expression was measured by RT-qPCR from equal volumes (equivalent of 4.2 ng original RNA/reaction). Cycle threshold (ct) values are shown on the y-axis and mRNA from all *toll* genes was detected in all tissues, with *toll-8* showing the lowest levels overall.
