# Supplementary material for: Elucidating the role of dsRNA sensing and Toll6 in antiviral responses of Culex quinquefasciatus cells
Source: Front Cell Infect Microbiol. 2023 Aug 30;13:1251204. doi: 10.3389/fcimb.2023.1251204 (PMC10499357; doi:10.3389/fcimb.2023.1251204)
Supplement: Supplementary file 2 [file DataSheet_2.docx]

**Toll protein sequences identified in *Culex tarsalis* genome (Main et al. 2021).**

>CtToll1

MGQRRRSWSEFQLVVALVVLLTHNLASSFPQHYDYVIGEELGGGGGAGLAAAKQSFKCPE

MTAISECSCMYASSDYEIQCPVTNPEITVKIKPDKYAQIQCYDRHDFGELPQLTLGDTEQ

VKIIHCTLPPHVSIRQYINFLGVKHMKEFWYQNYGKDLGVRLVRQHFDGMQDLEKLFLSS

GIEDIQPDLFANLSNLKWLVLRSNHVKLLHNVFDNLTNLTVLELGANQITELEPGLLKNQ

RKLLLLNLWRNQLRNITKESFRGAESLQQLDLSVNSMESLNPDVFELLPNLIELNLGFNR

FSSLPENLLSENRKLKDFKFINNQVPMRTLPSNFLSNLPQLTKVFLNRCNFVELPASLFR

GSSAILELDLSYNQLGALPEMLLRDQRELQTLNLAYNELEVLPEMLLENTGKLLTLRLSY

NRLRNLTSNIFASLKQLTELHLGNNQLHTIHVNTFSQTTALKRLDMQNNQLAFNDVSFVV

EEQNIGEGDGTPFQYLESLQVLNLRNNSIATVFRDWNVLNLKLKDLDLSYNNISTLNYHR

FEFLSEEIRVNLSHNRISEIDLSDMESIIGTSYNSDASKIFVDVSHNPLDCSCIFFFFAQ

YLQKEMNETVYKRIEIDTSNVQCAEPKNLRGTPVAQVKSEDVLCELDRPGTSIRRCPAGC

ACSTRRKDFAVIVNGTGRGLTQIPELPLPQSFGFRFIELHMDHNNITELPTNALPGYSNV

SKLYVANNAISELLPENLPVSLQTLDLTHNRLTSLNESVVEALNSSKLLTNLKLSGNQWR

CDCTASEMLRFVRNSFRKVSDISQVACNDGTSIEAIQIGDLCNENINTYITLSIVLSILG

LLIGLLTVLFYTYHMEIKIWMFTHNVFVWLVTEEELDKDKMYDAFISYSHKDEDFITEHL

IPTLEKEPMNFKTCWHLRDFMPGEMISEQIAKSVEDSRRTIVVLSNNFVQSEWAKMEFRT

AHLNSMSEKRVRVIIIIYGDIGDIEQLDSELKAYLKMNTYVKWGDPWFWDKLRYAMPHPP

TVKGLKRTGLIKNHIRSSVDDKLELIKPVPVTPPPLTTPPAEMNGTLSPSPFIISNGKTL

MNGNGYHGNGHLLNGHANGHVNGAYIINSNAKQSDV

>CtToll4

MKPFWLIAVFWLAQVGIPASGERLGGNVTELCPEECRSCRIDAARRSLTIDCSNSSLEEV

PLISREISLNSSSVTLDVRNNSIRNLPTIQDNPGFGVINKLLLDGNEFESIDVDSFHGNL

TSISMTYNKLHQLEDSFVDQILKSKTLRELVLLNNSWSCECPVAKQMIRLSTKYKQFRIP

TCTSGQSLINLNRSCITKIMFLMVLGSVLFTLTITFSALYIRHRHSIKVRLFSWQICLGF

VNHEEPDAAQLPFDAFISYSHRNDQFIVNELIPTLEKAPHSYKLCSILTQNFTPGDPIPT

QQFEQAIRSSRRTIIYITKQYLQSQWSLTSFRNTFETALTRSRTIVILAEDVEKFDELRT

SLRSKTLLERNDPFLWQKLLYAMPHRSAALMRKHRRDQKERTRKQAEMLRDVNRVREAKR

LADVVL

>CtToll6

MARSKLQPKSFAHLIRLKALSLEYCKIGKFGNDVLSGLSDLRNFTLRTHNINWPELNLEI

DPEVFVHSKNLEQLDLSMNNIWSLPDHLFCSLNNLRSLNISSNRLQDVNDLGFREKPIVK

DEVTTQNGTTHICNLDLEDLDVSKNHFVLLPASGFGMLKRLKLLKIHDNEISMVGDKALN

GLKELQILDLSSNKIVALPTDLFKDPAQSIQEIYLQNNSISVLSPGLFSKLEQLQALDLS

QNQLTSAWVNRETFSGLIRLVLLNLEKNKITKLESEIFSDLYTLQILNLRHNQLEIIAAD

TFSPMNNLHTLLLSHNKIKYLDAYSLNGLYALSLLSLDNNDLKGVHPEAFRNCSSLQDLN

LNGNELTQVPLALKDMRLLRTVDLGENSITVIEEPGFRGMNNLYGLRLISNNIENITRKA

FKDLPSLQILNLARNKIQFIEKGAFEPAISVQAIRLDGNLLTDIEGLFGAMPNLVWLNMS

DNRLSEFDYSQIPVHLQWLDLHKNELTTLGNRLGLDNQLYLQTLDASFNRLTKVTPTSIP

NSIEFLFLNDNQISHVEPHCFIHKTNLTRVDLYANQLIGLDIKALRLQPVPEDKQLPEFY

IGGNPFVCDCTIDWLQKINHVTSRQYPTINDIETVYCKLMYDRERAFISLIEAEPKHFLC

SYTTHCFALCHCCEFDACDCEMTCPNKCSCYHDNSWSTNIVECSAADYTDIPNNIPMDTT

EVYIDGNNLVELSGHSFIGRKNLKALYANHSNIEIIYNTTFIGLRRLTVLHLEHNNIQKL

YGNEFSALESLRELYLQGNKISYIEDHTFSELRKLEVLRLDGNRISSFEVWQLASNPYLV

EITLANNMWTCDCMFLNKLRVYLQSNQEKIVDASEISCIYNNSTSILKEKNGTKCTFRGE

GMSSIVHTQEIEDMLPLLLVATCAFVGFFGLIFGMFCYRKELKVWAHSSCFGSLCYKSGT

FVNEFDKDRLYDAYIINSLQDEHFVNQVLASTLENDIGFRLCMHYRDFNINTYIADTIVE

AVESSKRAILVLSKNFLYNEWTRFEFKGAIHEVLKRRRKLVIILYGDLPQRDLDADMRLY

LRTNTCIEWDDKKFWQKLRIALPHVKKSNCLNKRSAINIYATANEYNTAAAAGRPRLVGP

GNAGTLPAPGHARHESHSYATIGGNCPRNCDNYDTVSNCKYNTTQHERRLNDFSRKAADR

QHEYAVPSNCLLDTTHETYNTSCERIAGNETFECTSSSKYSTSSRGSSECSHSINGFHPP

PQQQVPNVLSTNFMVGGKAGAVGGDPAGGGFNTTTSASNNLNSNSSYNNSSRKPNNCPVA

DKGDRTAAVGTMGGDKNNFNDRRLPQAMWA

>CtToll7

MKIPENWGVSRLRRLQHLNLEYNNITELHGEALAGLSSLRTLNLSYNHLETVPAGLLAGS

RELREIHLQGNQLYELPRGLFHRLEQLLVLDLSRNQLSSHHVDNGTFSGLIRLVVLNLAH

NALTRIDSKTFKELYFLQILDLRNNSIGYIEDNAFLPLYNLHTLNLAENRLHTLDDRLFN

GLYVLSKLTLNNNLISIVERNVFKNCSDLKELDLSSNQLSEVPHAIRDLAVLRALDLGEN

QITYIENGTFANLNQLTGLRMIDNQIENITIGMFTDLPRLSVLNLAKNRVQNIERGSFDK

NLDIEAIRLDGNFLTDINGIFATLSSLLWLNLAENHLVWFDYAFIPSNLKWLDIHGNYIE

SLGNYYKLQEEIKVKTLDASHNRLTDIGPMNVPNSVELLFINDNHISTIHANTFIDKINL

ARVDLYANSLKKLQLHQLRVAPQPAEKQLPEFYLGGNPFECDCSMEWMQRVNNLTARQHP

KIMDLPNVECIMPHARGSPIRPIVSLKPKDFLCKYETHCFALCHCCDFDACDCEMTCPTN

CTCYHDQTWGTNVVDCGNQRAELAKPVPMDATEVYMDGNDYPELQNHAFIGRKNLKVLFA

NASKIITIQNRTFAGLTALEVLHLEDNAIQKIHGYEFENLALLKELYLQNNMISVIANNS

FAPLYSLQVLRIDGNRLTTIPMGQLQATQLQSLQALSLGRNYWSCRCRFMQELTSFVADN

AVIIQDMQDIYCVDEGIHRDLDFNVTASCSDYYAGSSVLPDRLSETYIFLLAAALIIACL

LVFVLVMFIFREPLRFWLFSRYGVRVFGPRCEDSEKLYDAIFIYSAKDAEYVARNIAAEL

ENGRPPLRLCLQHRDLSEDASHLQLLEASRASRRIVMLLSRNFLQTEWSRCELRRAVHDA

LRGRPHKLVVLEEVGVILEAENDVELVPYLKTATVNRIKRSDRHFWEKLRYALPVEAPYR

GNNYTIDHHERVKQTSGANGPAGVMFRQAPPAYCPEIDEANYSSATTATPSPRPSRRGMV

ELPQRPPSEHIYSSIDSDYSTLECENMVPGVHRPSATWRPGTGQNTMPHGHHVQAYLV

>CtToll8

MVHMYGLRLTENNIETIRKGTFDAMKSLQILNLSKNRLKRVEQACFDNNTNLQAIRLDGN

YLTDIAGLFTKLPNLVWLNISDNHLEVFDYALIPTGLQWLDIHANKIAELGNYFEIESQL

ALSTIDASSNQLTEITGSAIPNSVELLYLNDNLISKVQSYTFFKKPNLTRVDLFGNKITT

LDPNALRISAVPDDKPLPEFYIGGNPYQCDCNLNWLQKNNVDSRTQPKLMDLDSIYCKLL

YNRGRTYVPLVEALPNQFLCKYETHCFALCHCCDFYACDCKMECPARCTCYHDQSWTSNV

VDCSRAGYYEKLPDQIPMDSTQIYLDGNNFKTLSSHAFLGRKKLKILFLNNSNVEVISNR

TFYGLKELEILQLNHNQITELNGFEFVGLDRLKELLLQYNKIATIANQSFDHLHNLRILR

LDHNRIVEFNMWHLPKQLTDVRLAANSWSCDCEYVERFREYLKTYDFVKDRLKIKCATIT

LPGNDTALAVNGTTVTDPVRVVPDGGEGFLVYFDNSTTLCSGAIPLDNVINGNLTSRKTV

LSPAPIEGYIPLLVAALCGFSVIIIVTLIVFVFRQEMRVWFHSKFGVRLFYRNSDMDKNE

RDKLFDAFVSYSSKDEAFVAEELAPLLENGDPSYKLCLHYRDFPVGAYIADTIVQAVESS

RRTIMVLSENFIKSEWCRFEFKSAHHQVLRDRRRRLIVILLGEVPQKDLDPDIRLYLKTN

TYLQWGDKLFWEKLRFALPDVPNNQRRRQQQPPINMTHSNIRNQYQLTRTPNNRTSNQMM

MQQPLPPQPDPRLRPELPINVGQQQQQQQQQQSASTSAPARAPAAAAGSEPAAIVAEDGR

DPHMTCLRTVCDSVEIRLLLNFLYKVSR

>CtToll9

MAGYGEQDPYTSLTEGLELEDSVFDGLGNLSFLDLSHTKLLTSSARAFQHLAVRQLSLCY

TGIPILVGTMMRGTLQVLDISGNAGIASAIHHDKTDARGFNGNLEILVCENSTVKHLTWL

SGMTNLKVLMLASNNINQLLNTSFANMANLEILDLSSNHISNWHQRVFESNPNIYILDLS

DNNINVLTNEMLYDFANVQFLAIGQNSFVCHCLWREFIELAAKNAQSITCLLNSILNQLS

DPSKAQDEDDESASNSTTIKMLPERDFSLIERRTTTEGPALELLEVSSNEYNVLFRVVHS

YVSTIYDSSEKFLTSLEKYNLGRPPLRVYRKMIEARVSARFRSNICAEEPDAPADPPTVT

EDAEYVNPLYGLKIQIIDFDEDHYKCIDLDDSEFYLFEQERCTFDRSLLPNLNLPTYNGT

TANVIKFILIFFGFALIAFIIYISKWDHVKYFCIIVRNATILSMMKHKTEAKLGGGGGRK

ESLTSVVSSWMYDVFVSYSEQDRQWVLDELLPNMERTEDINVCLHERDFEVSHGSATVNH

TTYPFKKKILRSCRIHTRGACLLILRQFIPIMP

>CtToll10

MNLEFHPESFRGLTELKRLDLADNNIWSLPTDVFCPLFSLRQLNLTKNRLTDLSQLGFSD

WGNGPTAPGKACNTGLEVLDLSHNDILTLPDNGLSSLRSLNVLLIQDNLLTSLADRSFVG

LGSLKVVNMSSNKLVALPPELFQSPRELRQIYLQNNSLSVLAPGLLEGLDRLEILDLSHN

ELTSEWINRDTFAGLKRLVVLEISYNSLSKIDKHVFRELYSLQVLNLENNLIETIADNAF

SDLKNLVALTLSHNKLKRIDQHHFSELYVLNQLYIESNLIESMHSRALENLTNLNDLNLN

DNRLTEIPEGLGKLRFLKSLDLGKNRIVTVNNASFEGLEQLLGLRLVENRITNDSRDAFV

TLSSLHVLNLASNQIRHIDQSAFSSNPTIRAIRLDNNELEDISGVFTSLPALVFLNVSDN

QIRNFDYSHLPSSLEWLDMHQNNITELGNYYDLNNLHIKMLDVSFNRLATVDNKNVPDSI

ETLFLNNNVLEEVAAGTFLNKKNLEKVVLYGNYIKKLEIGALALTRVGDDKDMPQFYIGD

NPIHCDCTMEWLQGINKLSHLRQHPRVMDLDTVMCTMEHERGASLRPLMDLNSHDFLCQY

ETHCFATCHCCDFDACDCKMTCPDRCSCYHDHTWKTNIVDCGNADYTEVPEHIPMDATTI

YLDGNDLKQLGSHQFIGKKKLEVLYLNNSNIANVHNRTFNGIPSLRVLHMENNYIEELRG

FEFDQLTNLNELYLDHNAIGFVGEKTFENLKFLEVINLSDNKISEFSPWQALSAASETGS

LNKVSLEGNRWRCDCESLHKLQRWIRDVGGDFDINRMICADNRVVGDVISTCESRLEFVD

NEVAPPAVHRTVLMGHGGLIGGGYVPLLAAIVVAIIGTALIVALACVFRQDVRLWAHAKY

GVRLVKDPIVIASAKDQDQDKLYDSYVVYSIHDNEFVGRLLGAELQHYGYSVCLHHRDVH

SNTFLSDSLQSAADASKKIILVVSMNFLQNEWSQPQFRVALQSVIENIRPAYRRHKIVIV

LTAPVELVAMDPIMNLLIRTCTVACWGERKFWDKLRYALPDVNKDRTPKKLGDITRSPNL

RYTPAPTAMDQWCKISHPGVAVPVAVPQSTPSQSTCNTEDESSSASSQHYEAPMSQSHYN

MSRSSASLGHVYSTIPETPQMGRNGRAYFV

>CtToll11

ETQTLGNLAPARGSGATHYTLLLLLVVVVCCTLVTAARNNFLRHDDALPKGCTWTGAVLE

VSQEKRSDELQCKIKTITKTESLLANISSYQIDRIKSLKLECNDIMFFESSLESTTTPGN

FLGNLNSLLRLSIEYCKIKYIPAMAFSNMKVLKSLTLSTHNIDWSVMNLELHPDSFRGLT

ELKEMHLADNNIWSLPNEVFCPLYTLKVLNLTGNRLSDMSQLGLSDWGKGPIAPGKACNT

GLEVLDLSGNDITLMPDNGLSALRSLNALYLQNNLVKEIADRAFVGLGTLEILNLSNNKL

TALTPELFKSSRKIRQVHLQNNSLSVLAPGVFEGLDRLETLDLSRNQLTSTWIKRDTFAG

QVRLVVLNLGFNHLSKVDQHVFKGLYSLQILNLEHNAIELVADGAFSDLKNLHALFLSHN

RLRQIEPYHFSELYVLNQLILESNQIVYIHERAFENLTHLHDLSLNDNRLEEIPSGMKSL

KFLQSLDLGKNQITEINNSSFEGLEELMGLRLVDNQITEISRDTFFALSTIHVLNLASNR

IRHVDQSAFSSNPTLRAIRLDNNELEDVAGVFTSLTSLVYLNISDNNIGWFDYSHYPQSL

EWLDIHKNNISELGNRYDVGNWFQLKMLDVSHNRIRHINTSSFPKNIETLLLNNNQIEEI

APETFTGKENLVKVVLYGNHLRRLEMPSLALTLVPDTRTMPEFYIGDNLIHCDCSMEWLQ

RINELSYLRQYPQVKDLDSVMCTMEHERGELVRPLTEMKASEFLCKYESHCFATCHCCDF

DACDCKMTCPDRCSCYHDTAWESNIVDCGSAGLTTIPGKIPMDATDIYLDGNNFGQLESH

VFIGKKKLKSLYLNNSHIGEVNNKTFGGIPALTVLHLEGNGLERISGAEFEQLRELKELY

LDHNAIETVGNKSFYYQKSLEVLTMADNRLAELKPWELMPPGGTFRLISLSGNKLSCGCE

SIGKLVDWAERQFNETNGLSEFQCTNSKLVKDAIKEYYLPLLIAVLAGLVLTILLVTLVF

IFRNDVCLWAHSRYGVRICKDPLSTMERCEDNEKLYDGYLIYSSADADMGAADASRKLIL

FISVKFLQFEWSQAEFRAGLQSVLELIRPSRRKQKLILITSVPQSMLSMDPIMDILTRTC

TVIAWDDRRFWDKLRFAMPDIGKNSPVNKAPHRKAINIRYTPAPTNPSAPPDPTATWPKR

LNSEVCVQYPPHSPNHSTYNTEDELSTASSPQYEAPSLHHAMGPPYHHQNHIPLQQQQHL

HQSHHGQQVNHSPHLQHTFPHHMAPQPQHHPPQGYYRSGKSNGGGGSAANTLGHVYSTIP

ESQYNTHHSLRGGGAGDSNRPYFV
